# Understanding the policy discourse within the formulation of the 2013 Indian National Food Security Act

**DOI:** 10.1007/s12571-022-01267-y

**Published:** 2022-03-12

**Authors:** Karl-Axel Lindgren, Tim Lang

**Affiliations:** Centre for Food Policy, City University of London, England, UK

**Keywords:** Food Security, India, National Food Security Act, Policy Process

## Abstract

India was the third country in the world to enact into law a constitutional commitment to the right to food, following Brazil and South Africa. The 2013 National Food Security Act (NFSA) was the latest in a long line of post-Independence food policies aimed at tackling hunger. This paper explores the range of discourses among NFSA policy-makers, their views and disagreements, from drafting to the final Act. The research used mixed methods. Elite semi-structured interviews were conducted with 32 individuals who were either directly involved in NFSA formulation or food security specialist observers. Policy documents covering the period from before the Act and during the Act’s passage were critically analysed. Significant intra-governmental disagreements were apparent between two broad positions. A ‘pro-rights’ position sought to formulate a law that was as comprehensive and rights-based as possible, while a ‘pro-economy’ policy position saw the NFSA as a waste of money, resources and time, although recognising the political benefits of a food security law. These disagreements were consistent throughout the formulation of the NFSA, and in turn cast the Act as a product of compromise. Although there was broad consensus for a food security act, there was surprisingly little agreement exactly how that Act should look, what it should contain, and whom it should target. There was little consensus even on the right to food approach itself. The article contributes to the understanding of policy formulation in India specifically, and in developing countries in general, as well as to lend credence to the suitability of policy analysis to developing nations, otherwise normally grounded in Western traditions. The paper highlights a lack of cross-government cooperation in policy formulation, with the continued pressure of a short-term economic rationale undermining the policy goal of lessening hunger, despite some success.

## Introduction

India has had a long and troubled history with hunger, food insecurity and famines (Pal & Ghosh, 2007; Drèze & Sen, 2013; Mujumdar & Kapila, 2006). Despite significant strides in the reduction of undernourishment in the late 1990s, the 2000s saw a slow reduction in the prevalence of undernourishment estimated in 2011–13 at 217 million people, 17.2% of the Indian population (GNR, 2018). Nearly a fifth of the population are estimated to have been living with chronic hunger (Mander, 2012; FAO, 2014). This does not include the many millions more facing food insecurity without hunger through seasonal or temporary struggles to afford food, or as a direct result of an unforeseen crisis, such as occurred in the 2020–21 COVID-19 pandemic. This latest illustration of sudden food insecurity has left millions of people unemployed and unable to afford food (Mishra & Rampal, 2020; Bell, 2020). There have been many acute analyses of why this fragility persists, despite good intentions and many improvements. Explanations offered range from capacity problems to the gap between rights and realities, and to the minutiae of supply-demand mismatch (Gulati et al., 2012; Pritchard et al., 2014; Sharma & Gulati, 2012).

Given the scale of the hunger challenge, it is not surprising there is both a continuing concern for, and many approaches to, food security in India. These include: ecological stresses (Dasgupta & Sirohi, 2010; Gupta et al., 2015), nutrition and public health (Baviskar, 2018; Mander, 2011), political economy (Carolan, 2013; McMichael, 2009), and translating the right to food into legal entitlements. The latter highly influenced the Supreme Court of India, as well as policy-makers within the United Progressive Alliance (UPA) government that was in power from 2004 to 2014 (Aggarwal & Mander, 2013; Drèze, 2004; Deaton & Drèze, 2009). The presence of a rights-based approach was clear within the UPA, particularly in its first term 2004–09, wherein it passed a series of rights-based legislation, including the Right to Work (2005), the Right to Information (2005), the Right to Education (2009), and the Right to Fair Compensation in Land Acquisition and Resettlement (2013). The Indian National Food Security Act of 2013 (hereafter NFSA) was not only a direct response to continued hunger and food insecurity of the early 2000s, but a fulfilment of the UPA’s rights-based manifesto, albeit sparked by a Supreme Court case in 2001 that ruled that the constitution of India enshrined the right to food for every citizen (Drèze, 2004; Kent, 2005; Guha-Khasnobis & Vivek, 2007; Pillay, 2009).

India followed Brazil and South Africa in recognising the right to food as an inalienable right, placing it as one of only three countries to pursue a legally recognised right to food approach to address latent hunger (McDermott, 2012). The NFSA was thus the latest step in India’s long history of attempting to address food insecurity. These range from the complete overhaul of the agricultural system during the Green Revolution in the 1960s, to more specific programmes such as the Mid-Day Meal Scheme (MDMS), feeding 300 million children daily since 2005 (Guha-Khasnobis & Vivek, 2007; Pritchard et al., 2014; Mujumdar & Kapila, 2006). Since its inception as an independent, democratic nation, food security has been a core priority on both the federal and state levels, yet allegations of corruption and inefficiencies, as well as changes in policy mechanisms since the 1990s (such as moving from a universal to a targeted Public Distribution System (PDS)) has left many goals unfulfilled, and the production of sufficient food has not directly translated into food security for all (Gulati et al., 2012; Himanshu & Sen, 2011; Saxena, 2013; Sharma & Gulati, 2012).

The 2001 Supreme Court case spurred the ruling Indian National Congress (INC) party, as part of a broader centre-left coalition, to place a right to food bill, in an attempt to enshrine this right in a legal framework, central to their campaign platform for re-election in 2009. Food Security has historically been framed in India through a ‘donative discourse’ (Mooij, 1998) utilising a ‘service delivery’ paradigm (Menon, 2013): the government has seen itself as benevolent care-giver, delivering food directly through the Public Distribution System (PDS), and other food-related programmes such as the aforementioned MDMS in schools. Through the new framing offered via the NFSA, the ‘sharp’ key of a rights-based approach (Candel et al., 2013), citizens have the inalienable, legal right to food that must be addressed. Historically, the food security discourse in India has seen diverging claims, varying policy positions and differing interests, ‘framing’ food security in ways to promote specific interest (Candel et al., 2013). Following the move from a ‘universal’ to ‘targeted’ approach of the Public Distribution System (PDS), the main mechanism of food policy in India, there has been much debate and disagreement on how best to deliver food security.

This article specifically looks at the policy understanding and discourses between the policy-makers in and around the drafting of the NFSA. The focus here is on the policy formulation for the NFSA and the interplay between the policy-makers and institutions involved. While there has been analysis on how the NFSA has impacted grain markets (Sinha, 2013; Debnath et al., 2017), the environment (Sengupta & Mukhopadhyay, 2016), the process of implementation (Drèze, 2017; Khera, 2013), the details of the PDS (Pillay & Kumar, 2019), its right to food approach (Kishore et al., 2014), its role as a broad safety net (Narayanan & Gerber, 2016), and its impact on government expenditure, an aspect where much criticism was levied towards wastage within the PDS, which the NFSA greatly expanded (Acharya, 2013; Bhalla, 2011; Gulati et al., 2012), little has been written on the relationships of the actors, nor on how the NFSA developed through its various drafts, who influenced what, and why. The value in identifying the power dynamics among actors that are integral to policy formulation and the policy process, finding common ground with how policy processes are conceptualised in critical food policy analysis, affirms its applicability in different contexts. This article also contributes to critical food policy analysis by identifying themes and concepts found in the literature, particularly on actors and their relationship with power. The problems discussed regarding India also feature in other societies and contexts where not dissimilar effects of lobbying, civil society actions, and complex policy negotiations occur, despite vastly different socioeconomic and cultural starting points (Gilson & Raphaely, 2008; Clapp, 2012). The aim of this study was not to compare India’s food policy with Western democracies or other policy régimes, but to apply critical food policy analysis to the specific and research-rich Indian context.

Two primary research questions shaped the research:

- Who were the main actors (institutions, individuals, groups) involved in the policy formulation of the NFSA?
- What were the main points of contention and disagreements in the policy formulation of the NFSA?

These two research questions arose from a literature review of food security in India, where it remained unclear which main actors were involved in the immediate NFSA policy formulation, and how they related to one another. The other motive was to understand exactly what shaped the changes from the earlier drafts of the NFSA to the final Act, which differed substantially in terms of legally binding entitlements.

## Methods

This study used a multi-methods approach, utilising semi-structured elite interviews and policy document and parliamentary transcript analysis; these are methods widely used both in food policy analysis and wider critical public policy analysis (Rein & Schön, 1996). A central theme in critical policy analysis has been the role of the state as potential arbiter of the public interest (Cairney, 2012; John, 2012). Taking a more critical look at debates inside the state, sometimes out of sight, is one reason modern critical public policy analysis broke from and superseded the old managerialism of Public Administration as a government-serving discipline (Cairney, 2012). Modern critical food policy has adopted broader public policy analysis by applying a multi-actor, multi-sector, multi-level approach to research. Policy is made not just by those on high, with decisions percolating ‘down’ relevant structures, but also by the outcome of pressures from ‘below’ and ‘outside’ the state (John, 2012; Lang & Heasman, 2015). Today, let alone in the past, food governance is subject to fissiparous pressures. Asking participants and ‘inside-track’ observers and weighing what is said against recorded events is one way to explore policy dynamics.

In the present NFSA study, elite semi-structured interviews were conducted between 2015–2016. 32 individuals were interviewed, including food security activists, academics, specialists, government officials and politicians who were either directly involved in the formulation of the NFSA (policy-makers), or who were otherwise experts on food security or urban poverty. Interviewees were otherwise chosen for their involvement, based upon preliminary scoping inquiries, published sources, and eventual snowballing. Interviews were conducted in New Delhi, recorded and transcribed. Interviewees gave permission to be cited. Ethical approval was sought and given by City University London’s ethics system. Participants were recruited in two ways. Initial contact was made with people cited or known to be involved in policy processes, following policy documentary analysis. Thereafter a ‘snowballing’ process occurred with interviewees suggesting names and contact details. Snowball sampling is a model of interviewing that entails using existing contacts, or one singular contact, to help get in touch with other people of interest to the research (Noy, 2008). The starting point is usually a relatively small number of initial contacts that have access to the community or group being researched, and in turn can help establish links with other potential research participants (Geddes et al., 2018). Snowball sampling is considered the most widely employed method of sampling in qualitative research (Noy, 2008; Geddes et al., 2018). The initial starting point for this research was within the Right to Food Campaign, having established contact through earlier work with the organization. Policy document analysis generated names inside and outside the legislative process. Snowball sampling has its drawbacks, such as the inability to make wider inferences or to reflect a broader population, yet finds value in targeting specific groups and where there are low number of hard-to-reach participants, such as elite decision-makers (Geddes et al., 2018). Transcripts of the Lok Sabha debates on the NFSA were also reviewed. Care was taken to ensure people were interviewed who had close involvement and knowledge of the development of the NFSA. While media and private industry were also elements of the policy process, the focus of the article here is on those actively involved within government, as well as civil society members who were either directly involved outside government or viewed as experts on the subject (Miller & Boulton, 2007).

The study was conducted within a critical food policy conceptual framework (Clapp, 2012; Lang et al., 2009; Moragues-Faus et al., 2020; Vernon, 2007). This sees food policy-making as a flow of power relations, partly economic but also culturally, politically and societally framed, shaping discourse from primary production to final consumer and waste. Lang et al. (2009) argue that food policy analysis is an assessment of not only the relationship of evidence, policy and practice to the formulation and shaping of the food system, particularly in relation to the state, but also the role of policy-makers, proponents, experts and beneficiaries, and their taken positions, arguments, assumptions and expressed views of other sectors, mediated through the state, as part of the policy process. They argue that this gap between the passage of events and the official narrative should be closely scrutinised and theorised and is of core relevance to critical food policy analysis. Policy is thus subject to “power relations, conflict as well as consensus, irrational alongside logical thinking” (Lang et al., 2009). The research reported here set out to generate a better understanding of the policy processes behind the NFSA. In recent years, food policy creation has increasingly been discussed and researched as battles of interests over data, purpose and constituencies (Lang & Heasman, 2015; Clapp, 2017; Thow et al., 2018). Policy development is conceived as a jostling for position, with different perspectives, interests and ‘baggage’. The analysis presented here maps out the different actors and interest groups surrounding the policy – from those ranging from the ‘inner circle’, such as politicians, bureaucrats, and Members of Parliament, to those in the ‘outer circle’, such as advisory groups, academics and experts, with less attention on those on the ‘outside’, influencing but not directly involved in the policy-making, such as journalists, international organisations and private industry. Policy-making is informed and shaped by different visions, goals and interests. The policy arena is thus conceptualised as contested space, albeit with a structured bias; the intersection of competing issues and demands, a constant juggling of interests. The workings and the results of a policy may be presented as “seamless, rational” products, but are often produced less linearly or orderly as the final product would indicate (Lang et al., 2009; Lang & Barling, 2012).

Interviews, policy documents and parliamentary transcripts were analysed qualitatively utilising content analysis, particularly applying Ritchie & Spencer’s ‘framework’ theory (1994), developed specifically for applied policy research. Content analysis considers both the context of the documents and its content, interpreting what those documents contain and considering how they are affected by, and relate to, certain variables (Spencer et al., 2014; Grbich, 2013). While it encompasses many different strategies used in the analysis of text, its general approach is through systematic coding and categorizing large amounts of textual information to examine who says what, to whom, and with what effect (Bloor & Wood, 2006). The methodology of ‘framework’ theory builds upon identifying thematic frameworks, indexing the transcripts, charting the themes that emerge, and mapping and interpreting the data in a consistent and traceable fashion. Keywords, their repetition and frequency were an objective way to look at the themes that were emerging, while the subjective approach was also taken in utilising the research questions as a framework for the emerging themes and analysing the responses cautiously. A certain level of subjectivity in the analysis is to be expected, as long as one is clear in the development of the process and the data can be reproduced using the process. Coding was done by hand, the option to use NVivo was eschewed due to the inability of the programme to recognise syntax, nuance and inference (Krippendorff, 2013). 

There are multiple limitations of this article worth addressing. First, it is recognised that food security varies quite significantly between the different states of India (Gulati et al., 2012; Himanshu & Sen, 2011; Saxena, 2013), yet the NFSA is a federal-level policy, meaning the focus remains on the federal government, with only passing reference to states. While states are highly influential in the policy-making process, they are viewed here as actors on the legislative level, with representatives in the Lok Sabha and Raj Sabha. Secondly, the focus on government, civil society and academia as the core policy-makers means the role of media and private business are given much less attention. Thirdly, this article does not attempt to judge the achievements or the impact of the NFSA. The law has been gradually implemented throughout the country in the eight years since it passed through the Lok Sabha (India’s Lower House of Parliament). While initial data have shown an overall positive impact so far (Boss et al., 2021: Drèze, 2017; Puri, 2017), the long-term consequences of the NFSA are still to be established, with the impact of the COVID-19 pandemic on food security (Béné et al., 2021) yet to run its course, and the lack of an updated census since 2011 making final judgements even more uncertain (Boss et al., 2021).

## Findings

The findings showed that the development of the NFSA was a complex and branching process with several stages. While the judicial branch established the framework that would be adopted by the executive branch, the core of the shaping of the NFSA involved ‘outer circle’ policy advisors, experts and bureaucrats in the National Advisory Council (NAC) and Prime Minister’s Economic Advisory Council (PMEAC) and the ‘inner circle’ politicians and bureaucrats in the Prime Minister’s Office (PMO), Planning Commission and Ministry of Consumer Affairs, Food and Public Distribution. After a broad initial draft was accepted at the end of 2011, the NFSA was further shaped by the Lok Sabha Standing Committee on Consumer Affairs, Food and Public Distribution, who were heavily influential in the content of the final Act, if not its framework.

**Fig. 1:**
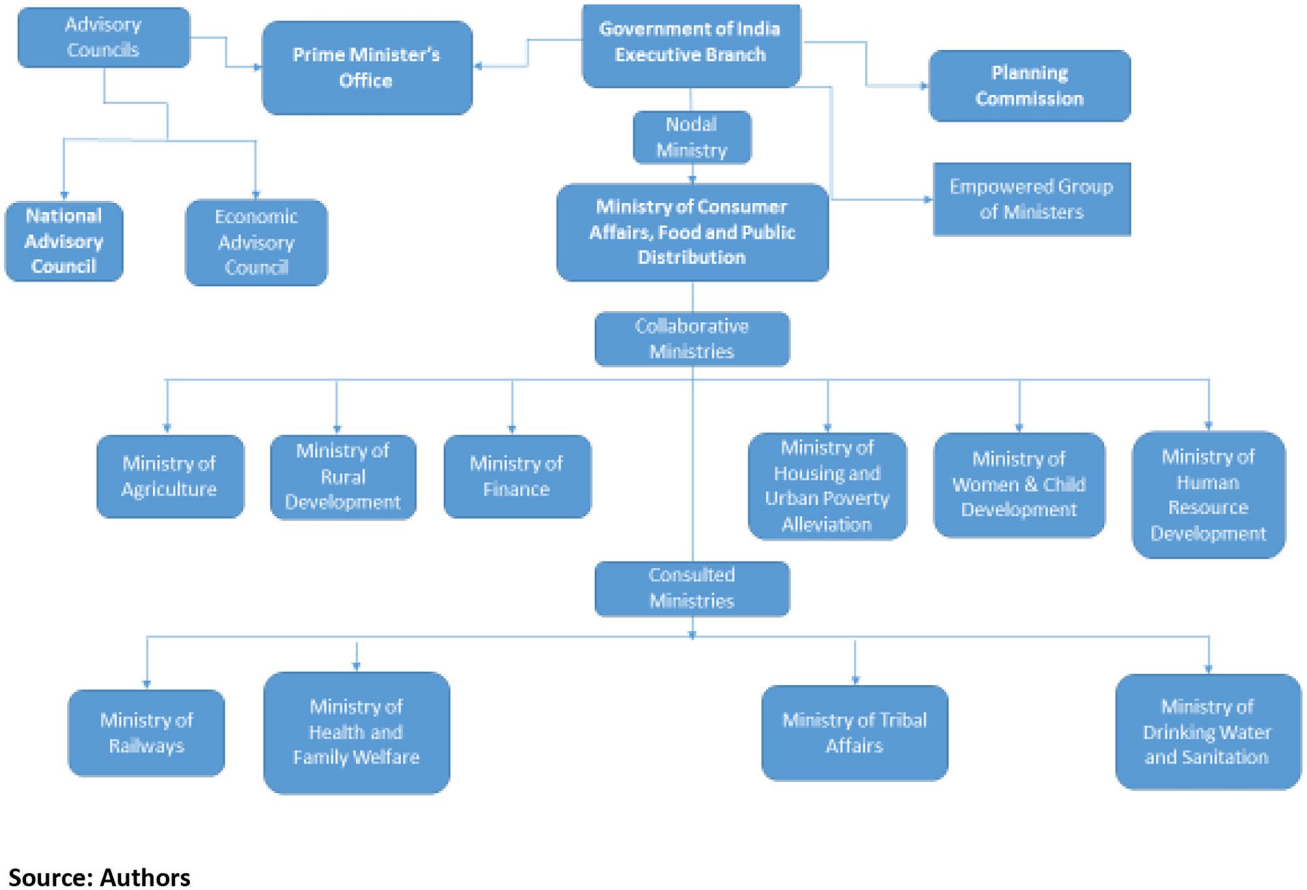
Executive Branch Stakeholders in the Policy Process of the NFSA, 2009-2013

The policy processes drew upon policy-makers from academia, civil society, bureaucrats, politicians, lawyers, experts from international institutions and Members of Parliament. Figure 1 below maps out the key institutions involved in the executive branch of the policy formulation stage of the NFSA. Table 1 summarises the development of the PDS, the core food distribution mechanic that the NFSA was designed to reform into a right to food context and expand.

**Table 1:** Timeline of Changes in the Public Distribution System, 1950-Onwards

Timeline	External Factors	Internal Factors	Changes
1950–1990	• Green Revolution expands agriculture, increasing yields and production • International discourse on food security pressure Indian government to ensure reduction of food insecurity	• Famines and starvation during British Raj, motivating politicians to ensure this does not occur again • Framework of Ration System from British Raj • Political discourse oriented towards ensuring food security for the poor	• Universalisation of PDS • Establishment of key Food Security institutions – Food Corporation of India (FCI) and Agricultural Commission of India (ACI)
1990–2013	• IMF and World Bank pressure through Structural Adjustment Programmes as consequence of loans • Public discourse critical of corruption, inefficiencies in PDS	• Political discourse re-oriented towards lowering government expenditure, cutting subsidies • Failure of universal PDS to sufficiently reach the most vulnerable enables alternate discourse for a targeted system	• Targeting of PDS • Establishment of Below-Poverty-Line (BPL) and Above-Poverty-Line (APL) Framework • Necessitates criteria for eligibility, further political differentiation of target population
2001-Onwards	• Right to Food Campaign, and broader public discourse, pressure government to deal with food insecurity • Failure of targeted PDS in dealing with food insecurity due to corruption and inefficiencies	• Supreme Court of India rule constitution ensures right to food • Political manifesto of ruling party to ensure re-election promises Right to Food Act • Tensions within ruling party between neoliberal economic perspective and rights-based perspective	• Establishment of National Food Security Act of 2013 • Expansion of PDS to two-thirds of population – 50% of urban and 75% of rural • Removes BPL/APL framework, but still necessitates eligibility criteria • No changes to food distribution mechanisms

Source: Authors

### The UPA-II Policy Platform – From Discourse to Law

The United Progressive Alliance (UPA) was a coalition of centre-left political parties formed in 2004, after no single party had won an absolute majority in that year’s election. The UPA was led by the Indian National Congress (INC), one of the largest parties in India and which has historically focused on a platform of raising living standards for the poorest segments of Indian society. This policy focus translated into a series of rights-based legislation passed by the UPA, including: The Right to Work, passed in 2005; The Right to Information, passed in 2005; The Right to Education, passed in 2009; the Right to Fair Compensation in Land Acquisition and Resettlement, passed in 2013; and the Right to Food, passed in 2013. According to JR, a key politician within the INC at the time, the ‘Right to Work’ was the ‘big idea’ that was forwarded in the election manifesto of the INC during the 2004 elections, and in the 2009 national elections the ‘big idea’ became the Right to Food. This ‘big idea’ was centred on the promise that malnutrition and hunger would be tackled, heralded as a comprehensive Food Security Act (Saxena, 2011; Mander, 2012). JR, who was a Minister during the UPA coalition times, elaborates in his own words:

“I had been entrusted with the responsibility of preparing the manifesto for the 2009 elections in May 2009. When you prepare a manifesto you look for one or two big ideas which can catch the public imagination. In 2004 the one big idea that caught the public imagination was the Right to Employment, what eventually became the National Rural Employment Guarantee [Act]. In 2009, what is the big idea that we can think of? Well, there are two big ideas, one of them the Right to Food, the other is the Right to Health.” – JR, former Minister.

Interviewees familiar with the UPA government indicated that there was a significant internal split regarding the NFSA and rights-based legislation, and this internal division was a central theme throughout the interviews regarding the policy processes. HM, a former member of the NAC, expressed the view indicating that this internal split was widely known by those who participated in the policy process:

“During UPA-1, because the Left was part of the alliance, there was a political incentive to see through rights-based laws. Sonia Gandhi represented the Left within the Congress and had a very strong ally in the Left parties. The second time around, in UPA-II, the political Left was out, and there were no political backers of the rights-based approach, just us outside the government who were advisors, policy-makers, bureaucrats, etc.” – HM, former member of the NAC.

The above quote from a member of the NAC, an unelected advisory body to the Prime Minister who were given the task to deliberate on and write the first draft of the NFSA, is indicative of the split between those on the ‘outer circle’ of policy-making with those on the ‘inner circle’. These advisors, policy-makers, etc. were not formally part of the government, yet had a significant role in shaping policy. This interplay between the NAC, the PMEAC, the PMO and the Planning Commission was repeatedly highlighted as a source of contention. BP, a former commissioner for the Supreme Court on the Right to Food, further clarified these divisions that were observed by these ‘outer circle’ policy-makers:

“Nobody wanted the NFSA. The Food Ministry was opposed, the PM was opposed, and the Planning Commission was also opposed. The bureaucrats, the government in power, the executive, the Prime Minister’s Office, they never believed in the NFSA.” – BP, former Supreme Court Commissioner.

Elite interviews with those intimately involved in the process elucidated the two factions within UPA-II – those who saw themselves as working towards a landmark rights-based legislation, and those who saw it as unnecessary, a misuse of resources and overtly costly.

The logic that seemed to be expressed through government policy documents (Rangarajan Expert Committee Report, 2011; Sivaramakrishnan & Singh, 2011) was that once the economy was at a sufficiently high growth rate, a ‘trickle-down’ effect would occur and the poorer segments of society would be able to afford food without the need for expensive, interventionist policies. Their primary objections to the NFSA were based on economic reasoning, taking cost and expense of a widely expanded Act as their primary concern (Rangarajan Expert Committee Report, 2011; Gulati et al., 2012). This division was spoken about in detail:

“[Sonia Gandhi] played a big role in the rights-based approach of Congress, and again in the second round of the government [UPA II]. However, the government was quite split and quite publicly so. The Planning Commission and the Prime Minister were speaking in a different language and the NAC speaking in a completely different language.” – DS, academic and activist in the Right to Food Campaign.

Perhaps due to the internal divisions within the government, the initial draft resulted from compromise between these two different ideological camps. JR, a Minister and key actor in the policy process of the NFSA, expands in their own words:

“From 2011 onwards, the debate [around the NFSA] was intensely political and there was a sharp division between the Prime Minister, the Finance Minister, the Agriculture Minister and the Deputy Chairman of the Planning Commission, all of whom were simply opposed to the Food Security Act, and on the other hand the NAC, including Sonia Gandhi, pushing the Act. Within the government there were only two of us who were pushing for the Act, myself and the Minister for Food, K.V. Thomas.” – JR, former Minister.

The development of the NAC draft was through compromise between the recommendations from the Prime Minister’s Economic Advisory Council (PMEAC) and the Planning Commission, changing the content, but not the framework, of the NFSA established by the NAC. Despite the PMEAC considering an ambitious food security act unsustainable due to the perceived need to drastically increase procurement to cover legal entitlements (Rangarajan Expert Committee Report, 2011; Gulati et al., 2012), the initial draft was ultimately heavily influenced by the rights-based framework set by the NAC. HM pointed out how unusual it was for the PMEAC to became involved in the drafting of the NFSA:

“[The development of the Act] was largely based on negotiations. Our [target] groups and formulations were retained, specifically women and children and vulnerable groups, except for the question of universal pension. Our draft went to the Prime Minister. The method was such that when the NAC concluded its deliberations, the chairperson, Sonia Gandhi, who was directly below the Prime Minister, went to him with the recommendations of the NAC… The Prime Minister was alarmed by the PDS component, so he did something unusual and unexpected, which was to refer to another council, the Prime Minister’s Economic Council [PMEAC], which is more conservative [than the NAC], and asked them for their views. The PMEAC expressed several reservations, turning around the draft quite substantially. They weakened the provisions around many of the aspects, including the women and child component. However, they did retain the vulnerable groups section to some degree, including community kitchens.” – HM, member of the NAC.

AS, a former official with the Planning Commission, summed up the role of the PMEAC neatly:

“The NAC had a set of proposals, Planning Commission had a different one, Rangarajan [the head of the PMEAC] sort of basically cut everything up. Basically, the final decision was that of the Prime Minister and the Finance Minister.” – AS, member of Planning Commission.

JG, a prominent development economist who was in the ‘outside’ circle, believed bottom-up activism and the insistence of rights-based legislation by Sonia Gandhi and left-leaning parties pressured the executive branch into introducing the NFSA to the legislative branch of government:

“They hated it – [the Prime Minister, the chairman of the Planning Commission, the Finance Minister] – hated all of these legislations, every single one of them. It was pushed by Sonia Gandhi certainly, but also by social movements, by political activism such as by the Left parties. The creation of the act was not something that happened because these guys at the top had a change of heart, or thought they were being good, it was pressure.” – JG, development economist.

Despite this apparent opposition within the INC, the supporters of the rights-based approach within the NAC managed to keep many of their desired entitlements during the negotiations, particularly for women and children, as stated by HM:

“The Prime Minister’s Office played a larger role in the NAC draft than any previous rights-based legislation. Many of the questions where they were involved was concerning the PDS. What we lost at the NAC stage of negotiations was including universal pensions. That was the major loss in terms of vulnerable groups, yet the rest of it was retained. Everybody’s focus was on the PDS, and on questions of coverage.” – HM, former member of NAC.

JD, a prominent development economist, echoes HM’s views that the predominant focus of policy-makers was to ensure legal entitlements through the PDS, with specific entitlements for vulnerable groups seen as extraneous to the main goal of an expanded PDS:

“The UPA was clear that they had made certain electoral promises. For them, the main thing was to activate this idea of legal entitlements through the PDS. They were never terribly keen on the rest [of the provisions]. They went along because these were obligations due to the Supreme Court order, so they said, ‘all right’. Some agreed because Mrs. Sonia Gandhi wanted the entitlement to be given, the rest resisted.” – JD, development economist.

CG, a prominent lawyer who was part of the Right to Food Campaign, views the policy process of the Act as being heavily influenced by a handful of actors who pushed through their vision, and were ultimately allowed to as it was seen as politically beneficial:

“The development of the Act was through a connection between some people in the Right to Food Campaign and some people in the UPA, and a desire by the Chairman of the UPA [Sonia Gandhi] to see things through. I think it had more to do with individuals in the Right to Food Campaign and individuals in the UPA government who pushed through quite a remarkable Act. The UPA felt that they would benefit politically from it, an internal calculation that if they come out with this social measure, even if they don’t implement it fully, it will benefit them politically.” – CG, lawyer.

Regardless of the true intentions behind the initial drafts of the NFSA, with actors pushing their own agendas, the NAC draft, and the negotiations between the NAC, the PMEAC, and the Planning Commission, were only the initial steps of the policy process. Once the NAC draft had gone through the Prime Minister’s Office, the suggestions of the PMEAC committee and the Planning Commission, the revised draft was then submitted to the Lok Sabha, India’s lower parliament. Tables 2 and 3 below maps out the changes made from the initial NAC draft of the NFSA to its final iteration, of which more will be explored in the next section.

**Table 2:** Iterations of the National Food Security Act, 2009–2013

Year	Draft	Key Aspects	Eligibility	Changes from Previous Draft
2009	National Advisory Council Draft	•Provisions for people living in/with: homelessness, destitution, emergencies, disaster zones •Community Kitchens for urban homeless and destitute •Starvation Protocol •Specific provisions for women and children •7 kg per person per month for ‘priority’ category at 3/2/1 rupees per kg of rice/wheat/coarse grain •4 kg per person per month for ‘general’ category at half of market price	90%	
December 2011	Government Draft introduced to Parliament	•Provisions for women and children •Community Kitchens for urban homeless and destitute •Starvation Protocol •7 kg per person per month for ‘priority’ category at 3/2/1 rupees per kg of rice/wheat/coarse grain •3 kg per person per month for ‘general’ category at half of market price	67%—75% rural and 50% urban	•Removed all provisions for the homeless, destitute, those living in emergencies or disaster zones •Reduced eligibility from 90 to 67% •Reduced ‘general’ category entitlements to 3 kg
January 2013	Parliamentary Standing Committee on Food, Consumer Affairs and Public Distribution Draft	•Provisions for women and children •5 kg per person per month at 3/2/1 rupees per kg of rice/wheat/coarse grain	67%—75% rural and 50% urban	•Removed ‘general’ and ‘priority’ categories •Uniform entitlements for everyone •Removed Starvation Protocol •Removed Community Kitchens •Extended provisions for children up to age 16
July 2013	National Food Security Bill/Ordinance	•Provisions for women and children •5 kg per person per month at 3/2/1 rupees per kg of rice/wheat/coarse grain •35 kg per household per month for AAY households, at 3/2/1 rupees per kg of rice/wheat/coarse grain	67%—75% rural and 50% urban	•Introduced provisions for the poorest of the poor – the AAY

Source: Authors

**Table 3:** Use of Contextual Keywords in the Lok Sabha NFSA Debates, 2011–2013

Keyword	Total Uses	Used in Repetition of Act	Used in Failed Amendments	Used Uniquely	Speakers who Used Keyword Uniquely
Farmer/s	70	0	7	63	19
Homeless	15	0	8	7	5
Labourer/s	6	0	3	3	2
Migrate	2	0	2	0	0
Migrant	0	0	0	0	0

Source: Author, Lok Sabha Transcripts

### Legislative Branch: Ministries and Parliament – Policy Finalisation

Once the NAC draft had gone through the PMO, the suggestions of the PMEAC and the Planning Commission, the revised draft was passed to the Ministry of Consumer Affairs, Food, and Public Distribution by 2012. As the nodal ministry, it was the minister’s responsibility to bring the draft to parliament for debate. As gleaned from policy documents and through the interviews, the ministry took the government draft as is, with very minor changes, and introduced it to the Lok Sabha, India’s lower parliament. K.V. Thomas was specifically highlighted by several interviewees as a key actor in supporting the NFSA:

“They got in K. V. Thomas. He was very good. He understood the importance of a Right to Food bill but he was also under a lot of pressure. The pressure was basically Montek [Singh], Manmohan [Singh], the leadership.” – KV, Activist in Right to Food Campaign.

“K.V. Thomas was the anchor [of the act].” – JR, former Minister.

Once introduced, the draft was passed to the Standing Committee on Consumer Affairs, Food, and Public Distribution. The Standing Committee consisted of 19 members of the Lok Sabha and nine members of the Rajya Sabha, and invited views, comments and suggestions from the general public, the nodal ministry, all states, as well as a select group of ministries, institutions and individuals who were considered to have the relevant expertise to comment on the draft act. Input was specifically sought from the Ministry of Agriculture and the Ministry of Rural Affairs (Standing Committee Report, 2013). The Standing Committee called for comments and inputs in the beginning of 2012 and convened in 2013 to discuss their findings and recommend changes.

The largest changes from the original NAC draft, and what would eventually become the final Act, were a result of the recommendations of the Standing Committee. Most of what the NAC had recommended had passed through the executive branch with minor changes, mostly in terms of language and phrasing. The Standing Committee recommended the removal of entire legally binding entitlements for specific target groups, arguing that there were difficulties in applying and enforcing eligibility for those target groups, as there were poor methodological frameworks with which to define them.

The Standing Committee changes were outlined by HM as severely weakening the Act in fulfilling food security:

“When the Standing Committee sat, the real destruction of the Act happened. They drastically eliminated [provisions for] vulnerable groups. That was the clueless blow in some sense, as they were supposed to be representative of all political parties and that’s where in the Right to Information Act and the MGNREGA things were improved. In this case the entitlements were further omitted.” – HM, member of the NAC.

Nobody on the committee was able to be interviewed during the research, so the Standing Committee’s intentions outside of the wording of the policy papers remains elusive. However, the crux of the changes lay in the Standing Committee’s belief that “it was the utmost importance” that the NFSA was to be a “simple yet effective framework of the Public Distribution System ensuring food security to the people of India” (Standing Committee Report, 2013, pg. 24). As such, the specific target groups, such as the homeless, the destitute and those living with starvation, were perceived to be complicating an already broad bill and that keeping those entitlements in “would be difficult for the administration to identify destitute and homeless persons who may be given such benefits under the provisions of the Bill. Further, there is a risk of breaking the social fabric as non-earning members of the family may be pushed out of homes to feed for themselves.” (Standing Committee Report, 2013, pg. 104).

The recommendations by the Standing Committee in this regard are almost wholly based on the input of a singular member of the Lok Sabha with the INC, whose comments were copied without change for the Report's final recommendations. All other comments noted in the report suggest ways of improving the definitions and identification of the destitute and homeless, or an expansion of the existing entitlements to be more comprehensive. However, it seems that this singular recommendation was a sentiment shared by the Standing Committee.

JD, a prominent development economist who was a member of the NAC, did not agree that the Standing Committee changes were merely weakening the Act, but making it more practical and manageable:

“The government was waiting for the Standing Committee to give their report, but for whatever reason wasn’t moving. It was only after they sent it back that it went to the Parliament, and by that time they were in a hurry because the next election was coming up, so they accelerated the whole process.” – JD, development economist.

After the deliberations of the Standing Committee, the revised draft was reintroduced to the 15th Lok Sabha in 2013. The transcripts of the debates were analysed for keywords to better understand the context surrounding the NFSA, what topics were and were not discussed. Out of the 545 members of the Lok Sabha, only 23 MPs were active in the debate around the NFSA outside of mere reiteration of the words of the Act. This may be common among legislative debate, in India or otherwise, but is worth noting.

The keyword “Farmer/s” was chosen to reflect rural concerns; “Farmers” were mentioned 70 times, despite the NFSA not once mentioning farmers specifically, with seven of those mentions in the context of unsuccessful amendments, mentioned by 19 individual speakers. To reflect concerns for vulnerable groups mentioned in the earlier drafts of the NFSA, the keywords “Homeless”, “Labourer/s”, and “Migrant/s” were chosen, to reflect the most vulnerable urban groups that were identified by the NAC. The transcript analysis showed that “Homeless” were mentioned 15 times, eight of which were in relation to removed entitlements, and by only five speakers, while “Labourer/s” were mentioned six times, three of which were in relation to failed amendments, and by two speakers. Lastly, “Migrants” were not mentioned at all, with “Migrate” only appearing twice, both in relation to “Labourers” within a failed amendment.

DT, an MP from West Bengal who supported the UPA-II coalition until 2012, was one of the only MPs who spoke strongly against the removal of the entitlements for vulnerable people in the transcript:

“The NAC had detailed chapters in their draft version of the NFSA on dealing with the needs of the destitute and most marginalized sections of our country, including the urban homeless, people affected by starvation, out of school children, destitute feeding programmes, community kitchens, emergency feeding programmes and so on. I am deeply saddened to note that all of these progressive parts of the NAC draft have been removed from the Bill that has now been listed in Parliament, even though they were included when the NFSB was tabled in December 2011.” – DT, Member of Parliament.

RCD, MP from West Bengal and member of the Communist Party of India (Marxist), introduced an amendment that would provide access to the PDS for migrant labourers without a set address, including both landless agricultural labourers and urban labourers in the unorganised sector, which was defeated after being put to vote. This amendment, which was introduced to the House twice, included the following entitlement:

“(1B) All homeless persons shall be entitled to affordable meals at community kitchens, in accordance with such scheme including cost sharing, as may prescribed by the Central Government” – Amendment No. 88, Lok Sabha NFSA Debates.

These amendments were voted down each time. The first vote was done in division of the house, showing that there were 144 Ayes and 241 Noes for the amendment. While the quoted entitlement was only a smaller part of the larger amendment, the overall purpose of the amendment was to reintroduce the specific entitlements that had existed in the NAC draft. A second division was done for a more specific amendment, which was to insert the following line:

“Foodgrains shall be provided free of cost by the Central Government in case of entitlements of destitute persons, homeless persons and people living in starvation or conditions akin to starvation.” – Amendment no. 283, Lok Sabha NFSA Debates.

This division, for a much more specific amendment, showed that there were 109 Ayes and 235 Noes. This indicates that there was no broad backing or desire for the re-introduction of specific entitlements for vulnerable groups among the MPs in the Lok Sabha.

As such, there was continued opposition to the bill from a variety of different sources. One part was opposition from states that had implemented universal PDS, such as Tamil Nadu, who feared that the standardisation of the PDS through the NFSA would weaken their own PDS; one part was due to economic concerns from certain parties, in terms of government expenditure, such as the-then major opposition party, the BJP; and one part was due to political and ideological differences from opposition parties to the UPA, who saw the NFSA as far too expansive. Despite these oppositional voices, there was no real concerted pushback to the NFSA in its totality. This was due to it addressing hunger and malnutrition, a policy goal that cut across state and party lines.

## Discussion

The policy arena which created the NFSA involved multiple institutions, with significant key actors contributing to substantial changes in policy formulation. India in this respect confirms what studies of other policy processes have found (Parsons et al., 2018; Thow et al., 2018). The importance of the role and involvement of these institutions and actors came down to the perceptions and the narratives shared by the interviewees and careful reading of policy documents. The findings indicate that the UPA and UPA-II governments involved non-governmental policy experts and academics in the policy process to help shape rights-based legislation, predominantly through the NAC, as well as through consultations and a call for recommendations by the Standing Committee on Consumer Affairs, Food and Public Distribution. While this involvement lessened once legislation reached the parliamentary stage of the Lok Sabha, the ability to communicate with non-governmental groups led to very ambitious legislation that had potential to raise the living standards for tens of millions of people.

However, during the period that the NFSA was being discussed and drafted, the UPA-II government was hit by a series of scandals, with corruption rife on many levels (Baru, 2014). The present study found that academics and activists were of the opinion, in hindsight, that their involvement in rights-based legislation, helping shape the discourse around rights and poverty relief, was a way to ‘legitimise’ the government and obfuscate its unwillingness to implement the legislation and make tangible strides towards reducing poverty, a policy thorn still stuck by a mindset of ‘donative reality’ or charity (Menon, 2013; Mooij, 1998; Schaffer, 1984). Instead of challenging the historically paternalistic approach, the rights-based approach was instead co-opted, its language used to disguise how the actual governance had not substantively changed (Mander, 2012). The NFSA, specifically, came at the tail end of the UPA-II government and came too late for the UPA-II government to implement; it was left to future governments to use and oversee.

The legislation itself was also heavily delayed, sitting in the Lok Sabha for at least two years. The Lok Sabha debate transcripts show that internal political opposition to the Act came in the form of MPs from Tamil Nadu and Chhattisgarh, who were otherwise allied to the ruling coalition, as those states had implemented universal PDS and were worried that the NFSA would limit their mandate. The bill was otherwise broadly supported, regardless of political affiliation, as it was viewed as something that would help the poorest segments of the population and thus politically difficult to oppose. Thereby the consensus frame over food security emerges (Mooney & Hunt, 2009). Who could be against legislation so deeply embedded in independent India’s political mindset? Despite the change in language, the actual content and design remained within the framework set by existing policies, although the possibility of expansion of entitlements, beyond the PDS and for women and children, were curtailed.

The Lok Sabha transcripts, and the interviews cited here, show that the aim of feeding the populace was never in question, and never challenged; even the Bharatiya Janata Party (BJP), the major opposition party, was in favour of the NFSA. Where the disagreements lay were exactly in the ‘fractured consensus’, to use the term of Maye and Kirwan (2013), over the best way to address food security in a country where continued hunger is normalised. Farmers, their plights, and the rural problem were frequently invoked, and the debates that raged were more to do with states’ rights and appropriate compensation than on the value of food security in itself. Two broad camps could be identified as emerging from the tensions and disputes that underlined the policy process – the embedded, dominant frame of ‘free market’, economy-first perspectives, as Davies (2014) and Candel et al. (2013) argued is common among government agencies, and the ‘counter-frame’ of the left-wing of the INC, as well as Leftist and communist MPs in the Lok Sabha, the Right to Food Movement, and the academics and civil society activists who found themselves at the heart of the initial draft. It should be stressed that India has never had a dominant frame of the free market, even after liberalization in the 1990s, yet a growing number of economists and politicians, particularly within the INC party, were of the mindset that India should reduce public expenditure and cut costly aid programmes. The embryonic NFSA was an opportunity to push the alternative perspective they had been championing for many years. Several of the interviewees believed that the four years the NFSA took to go from initial drafting to final Act, reflected how many prominent INC members had little faith in the NFSA’s prospects, seeing it as a waste of money, a largely ideological undertaking, and a strain on resources. This divide within the UPA-II government was mentioned repeatedly by different interviewees, highlighting other conceptual differences. Some policy-makers wished to prioritise economic growth and minimise government involvement, while other policy-makers argued that the entire platform of the UPA and UPA-II government was to provide for the poorest of the poor, and that the only long-term policy approach to ameliorating poverty lay in rights-based programmes providing work, food, education and information.

This discourse runs through the NFSA’s policy formulation, between policy-makers who seemed primarily motivated by the plight of the poorest, those living on the margins of society and who suffer from food insecurity, and policy-makers who were more focused on economic growth and lessening the burden of government expenditure on costly programmes (Sen, 1981; Drèze & Sen, 2013). During the first UPA, several rights-based programmes were drafted and passed in a year, such as the Right to Work and the Right to Education, but by the second UPA, only the NSFA was touted as a rights-based Act and purely derived from a campaign manifesto promise. The INC itself was divided on the topic of economic growth vis-à-vis poverty alleviation programmes, and the shedding of left-wing parties from UPA to UPA-II shifted the ruling coalition towards a broader economic growth platform and away from the original poverty alleviation platform that had proved to be popular with the electorate when they first came to power (Drèze & Sen, 2013).

Despite these political shifts, the NAC and the initial government draft did contain provisions for a starvation protocol, as well as for the homeless and migrants, reflecting those policy-makers involved at the early stages of the policy process, specifically the NAC stage, who had recognised the need to provide food security provisions for vulnerable groups. Despite the disagreements among central policy-makers within the ‘inner circle’ of government, there had been significant provisions included in the first draft of the Act for vulnerable groups. These provisions were ultimately removed by the Parliamentary Standing Committee for Public Distribution, Food and Consumer Goods. The Lok Sabha transcripts show that specific entitlements for vulnerable groups was mostly neglected in the debate, and their position of power to ultimately decide over the Act led to huge excisions that would have specifically aided vulnerable groups. Furthermore, the language used in the Standing Committee report justifying the excision of entitlements for urban vulnerable groups was entirely drafted by one INC MP.

This study presents further evidence that broad support for food security and ending hunger does not necessarily translate into a unified vision on how to accomplish this. The embedded, dominant frame of ‘free market’, economy-first perspectives continue to dominate the global food security discourse (Davies, 2014; Candel et al., 2013), in turn indirectly influencing India’s own food policy, which undermines the stated policy goal of lessening hunger, further compounded by the lack of inter-government cooperation in policy formulation. Despite the focus on ‘growth-led development’ (Sen, 1981; Drèze & Sen, 2013), a consistent and vocal grass-roots movement and key policy-makers within government receptive to novel policy approaches can open policy formulation to new opportunities. The embryonic nature of the NFSA highlights the opportunities that can occur when experts and academics are invited in to help formulate policy, garner the support among key policy-makers within the government, and push a strong counter-narrative, potentially leading to genuine policies that seek to address hunger and food insecurity.

The importance of this deep difference in ideological framing assumptions is perhaps no surprise. Critical food policy thinking has long shown that what might appear to be above ideology or politics – the case for feeding all citizens healthily, equitably and sustainably- is fraught in reality, with tensions, disagreements, and compromises of the actors involved (Lang & Barling, 2009; Coff et al., 2008; Clapp, 2012; Carolan, 2013; Nestle, 2013; George, 1978; Vernon, 2007; Shaw, 2007; Drèze et al., 1995). The present study not only highlights that India’s internal policy formulation shares similarities with how food policy is negotiated in developed countries, but that the right to food was more easily made as discourse than as implementable entitlements and policies. Without the opportunity for citizens to claim a right to food, the impact of the rights-based language is limited (McDermott, 2012).

## Conclusions

As conceived by critical policy analysts, this study confirmed that the NFSA policy-makers were not one homogenous group. Understanding the tensions and different policy goals and desires of the different actors is key in understanding how policy was shaped. Despite the inherent ‘messiness’ and unpredictability of policy-making, the findings have shown an insight into how different policy groups and institutions had controlling stakes in the drafting of the Act at different stages. It was clear that there was not necessarily a homogeneity or agreement even within institutions, with different competing interests. The findings show that key actors had disproportionate influence in removing provisions for vulnerable groups late in the policy process, removing the potential to address specific food insecurity needs.

The study further shows that there were significant tensions throughout the drafting process, particularly between policy-makers in the early stages that can be broadly grouped as ‘pro-rights’, who argued for a generous food security act with substantial entitlements for vulnerable groups, and what can be broadly labelled a ‘pro-economy’ group, who viewed the entire exercise as economically wasteful and an increased burden on state expenditure. Despite these disagreements, the ‘pro-rights’ group achieved substantial goals in terms of language and framework, with most of the content drafted by the pro-rights camp reaching the legislative branch. Here, however, it was heavily excised by Members of Parliament, whose motivations remain frustratingly oblique. While the framing and language of the NFSA is almost wholly based on the initial draft of the NAC, its content was ultimately a compromise between these groups.

Interestingly, the points of contention were not around the right to food – this was broadly accepted – but around the details of how exactly a right to food act would look like. Due to the consensus frame of food security (Candel et al., 2013), no actor explicitly disavowed or disagreed with its existence, but instead sought to influence its development as a policy in other ways: the politicians, activists, academics and bureaucrats who were pushing for comprehensive, rights-based entitlements, and the politicians, bureaucrats and members of parliament who were looking at reducing costs, ‘streamlining’ the Act and removing entitlements that were outside its two core aspects of the PDS and nutrition for women and children. This shows that despite broad support for ending hunger, there is no singular vision that is easily rallied behind and is thus vulnerable to exploitation, being a broad concept that is hard to disagree with.

Early drafts contained comprehensive entitlements for vulnerable groups until the subsequent excisions of entitlements in the Lok Sabha. The implications for policy highlight how a short-term economic rationale undermines policy goals of ameliorating hunger, while the language of rights and ‘food security’ obfuscate such interests. The tensions summarised could be dismissed as of little surprise. It is important, however, to acknowledge the politics of India, the most populous democracy, recognising the enormity of food security. Critics might argue that the NFSA reprises the old themes of deficit rather than rights; or markets versus subsidies; or the rural over the urban. Further considerations that were not even mentioned or raised, such as incorporating sustainability or agricultural concerns, and not just direct food distribution, into its frame of reference, could well be levelled as a criticism. It remains true, nonetheless, that the NFSA, for all its faults, shows that without legislation, policy-making remains rhetoric. Many lessons for those pushing for reforms from outside India’s parliamentary system arose, such as the need to have clear strategic alliances inside and outside, from the federal to state and local levels. Nonetheless, the NFSA remains a landmark moment. Future research looking at the role of private enterprise and the media on shaping policy would also help expand the insights uncovered in this study, as well as a further evaluation of the outcomes of the NFSA. Preliminary studies have shown that implementation has increased coverage, reduced administrative errors, and seen positive steps towards reforms, albeit not without its challenges (Boss et al., 2021; Drèze, 2017; Puri, 2017). The changes done at the legislative stage, for example, reflect the influence of key policy-makers in the legislative branch, which merits further research. Even with perfect implementation (in itself a massive issue in India), the NFSA would not adequately address the food insecurity of millions of people that find themselves outside the system.
